# Analyzing the Effect of Vaccination Over COVID Cases and Deaths in Asian Countries Using Machine Learning Models

**DOI:** 10.3389/fcimb.2021.806265

**Published:** 2022-02-08

**Authors:** Vanshika Rustagi, Monika Bajaj, Tanvi, Priya Singh, Rajiv Aggarwal, Mohamed F. AlAjmi, Afzal Hussain, Md. Imtaiyaz Hassan, Archana Singh, Indrakant K. Singh

**Affiliations:** ^1^ Molecular Biology Research Lab., Department of Zoology, Deshbandhu College, University of Delhi, New Delhi, India; ^2^ Department of Computer Science, Deshbandhu College, University of Delhi, New Delhi, India; ^3^ DBC i4 Centre, Deshbandhu College, University of Delhi, New Delhi, India; ^4^ Department of Mathematics, Deshbandhu College, University of Delhi, New Delhi, India; ^5^ Department of Pharmacognosy, College of Pharmacy, King Saud University, Riyadh, Saudi Arabia; ^6^ Centre for Interdisciplinary Research in Basic Sciences, Jamia Millia Islamia, New Delhi, India; ^7^ Department of Botany, Hansraj College, University of Delhi, Delhi, India

**Keywords:** linear regression, machine learning, COVID-19, polynomial distribution, OLS regression, Support Vector Machine (SVM)

## Abstract

Coronavirus Disease 2019 (COVID-19) is spreading across the world, and vaccinations are running parallel. Coronavirus has mutated into a triple-mutated virus, rendering it deadlier than before. It spreads quickly from person to person by contact and nasal or pharyngeal droplets. The COVID-19 database ‘Our World in Data’ was analyzed from February 24, 2020, to September 26, 2021, and predictions on the COVID positives and their mortality rate were made. Factors such as Vaccine data for the First and Second Dose vaccinated individuals and COVID positives that influence the fluctuations in the COVID-19 death ratio were investigated and linear regression analysis was performed. Based on vaccination doses (partial or complete vaccinated), models are created to estimate the number of patients who die from COVID infection. The estimation of variance in the datasets was investigated using Karl Pearson’s coefficient. For COVID-19 cases and vaccination doses, a quartic polynomial regression model was also created. This predictor model helps to predict the number of deaths due to COVID-19 and determine the susceptibility to COVID-19 infection based on the number of vaccine doses received. SVM was used to analyze the efficacy of models generated.

## Introduction

Pandemics have been created throughout history; pandemics have been caused by new strains of viruses, such as influenza, increasing sickness, death, and destruction in the countries. Some of the acknowledged pandemics include the Spanish flu (1918), the Asian flu (1957), the Hong Kong flu (1968), and the Swine flu (2009), each varying in the morbidity and mortality rates (Akin and Gözel, 2020). Coronavirus, or SARS-CoV-2, which causes Coronavirus Disease (COVID-19), first surfaced in the United States with COVID-19 outbreaks in Wuhan, China, during December 2019 and spread throughout the globe in less than a month like a forest fire. The first confirmed case in the United States was reported on January 20, 2020, (Holshue et al., 2020). As of May 14, 2021, there are 161,846,189 instances of COVID-19 and 3,359,004 persons who have died due to this disease worldwide (Worldometer, 2021). The coronavirus disease 2019 (COVID-19) pandemic, which is caused by severe acute respiratory syndrome and has spread to 220 countries, has resulted in over 63 million laboratory-confirmed cases and over 1.4 million deaths [World Health Organization (WHO)], causing widespread social and economic disruption.

Coronaviruses are members of the Coronaviridae family, which is part of the Nidovirales order (Enjuanes et al., 2006). Coronaviruses are programmed to successfully adjust to a wide range of hosts (Mammals and Aves) and tissue tropism and have the ability to adapt to novel environments through mutation and recombination (Graham and Baric, 2010). Coronaviruses are positive-stranded RNA viruses. The RNA-dependent RNA synthesis in Nidovirales involves a discontinuous step during the development of sgRNA mRNAs (Enjuanes et al., 2006). A helical capsid consisting of nucleocapsid proteins encases the viral genome. The three major structural proteins found in the virus include: the Membrane protein (M), and the Envelope protein (E), both assist in virion assembly, while the Spike protein (S) aids in the entry of the virion into the host cells (Li, 2013). SARS-CoV-2 has many copies of the S glycoprotein on its surface, which gives the virus its unique crown form. ORF1 3′ is dependent on the S protein’s ectodomain's tropism-altering interchangeability and the inherent capability of coronavirus-targeted recombination (de Haan et al., 2008). The S glycoprotein of SARS-CoV-2 is divided into two domains: an amino-terminal S1 domain-containing 200-amino acids receptor-binding domain (RBD), and a carboxy-terminal S2 domain-containing a potential fusion peptide, two heptad repeat (HR) domains, and a transmembrane (TM) domain that forms a trimer (Bosch et al., 2008).

The S1 and S2 subunits of the S glycoprotein are important for the interaction between host and virus, which impacts the range of the host (Naqvi et al., 2020). An investigation also discovered that spike protein had several alterations in its structure. Destabilizing mutations have been discovered in the SARS-CoV-2 replication machinery enzyme and S, E, and N structural proteins which cause an increase in infectivity rate (Mohammad et al., 2021). It has been established that an elegant coronavirus reverse-genetics technique can effectively introduce mutations in SARS-CoV-2 genomic regions. The cellular proteases cathepsins, HAT (human airway trypsin-like protease), and TMPRSS2 (transmembrane protease serine-2) are responsible for penetration of SARS-CoV-2 within the host cell and splitting up the spike protein (Upadhyay et al., 2020). The angiotensin 1-converting enzyme 2 (ACE2) molecules on the host cells act as a receptor for SARS-CoV2 (Li et al., 2003). This initiates the entry of the virus into the host cell, which in turn leads to COVID-19 infection. Even though a lot of different research has been conducted on COVID, we are still unsure of the mutation that causes the COVID genome to mutate. Any information on the source or rate at which the COVID virus' genome is altering will be extremely useful in preventing the virus' future development (Asrani et al., 2020).

COVID-19 symptoms include fever, cough, chest tightness or pain, tiredness, and sore throat. However, asymptomatic infections have also been reported. In some studies, it was found that during the infection, the white blood cell counts of the patient were found to be normal (71.4%) or decreased (28.6%) in nearly 70% of the patients, and lymphocytopenia was found in 50% (7/14) of them (Su et al., 2020). Dr. Sakoulas estimated that 80% of COVID-19 participants will not require medical attention, 15% may require non-ICU medical care, and 5% may require ICU stays (Dr. Sakoulas, 2020). Pneumonia puts patients' lives in jeopardy. COVID-19 appears to be milder in children than in adults. Approximately 90% of pediatric patients are diagnosed with asymptomatic, mild, or moderate disease. According to the statistics, one-quarter of COVID-19 deaths occurred in older adults aged 70–79 years. Up to two-thirds occurred in those over 80 years, regardless of the incidence of the disease or the completeness with which deaths were recorded across nations (Calderón-Larrañaga et al., 2020).

The selection of appropriate specimens is crucial for identifying COVID-19 infected individuals in the majority of cases. The nasopharyngeal swab is commonly used for diagnosis. Still, lower respiratory tract specimens such as sputum and broncho-alveolar lavage (BAL) may also be used to increase the chances of detection (Loeffelholz and Tang, 2020). Antibodies against SARS-CoV-2 are detected using an enzyme-linked immunosorbent assay (ELISA) based on the recombinant nucleocapsid protein of SARS-CoV-2 in patients with confirmed or suspected COVID-19 within 3–40 days after symptom onset (Xiang et al., 2020). An immunological test is essential in establishing whether or not a person has COVID-19, but only in the early stages of infection (Asrani et al., 2021a). COVID-19 serodiagnosis with IgM and IgG ELISA offers high sensitivity and specificity for COVID-19 detection, which improves the accuracy of diagnosis. Researchers discovered that the sensitivity and specificity of IgM detection in confirmed COVID-19 patients was 77.3 and 100%, respectively, and that the sensitivity and specificity of IgG detection were 83.3 and 95.0%, respectively. The sensitivity and specificity for IgM in suspected COVID-19 cases were 87.5 and 100%, respectively, and 70.8 and 96.6% for IgG. As a result, the increased specificity of IgG and IgM detection improves accuracy and may aid in diagnosing COVID-19 patients (Xiang et al., 2020). Serological testing is crucial in this pandemic to discover active cases. The pandemic due to the coronavirus (COVID-19) has underlined the necessity for precise and speedy COVID-19 detection, and real-time RT-PCR is the most often used method for coronavirus identification (Asrani et al., 2021b). Molecular testing remains the “gold standard” for relevant case diagnosis (Xu et al., 2020). Other approaches that have been developed include loop-mediated isothermal amplification, clustered regularly interspaced short palindromic repeats (CRISPR), and multiplex isothermal amplification followed by microarray detection (Loeffelholz and Tang, 2020).

In this pandemic, the most prevalent care techniques for severe cases include artificial breathing, ICU admission, and supportive care (such as bed rest, adequate nutrition, avoidance of dehydration, electrolyte and acid–base balance maintenance, antibiotics, and isolation of patients suspected or proven to have the infection), according to the WHO guidelines (Liu et al., 2020). Mass media and Public health communication contribute significantly to the pandemics from SARS in 2003, H1N1 in 2009, and MERS in 2012 (Anwar et al., 2020). At the time of COVID infection, media coverage of hardships, lockdown, and quarantine generated stress and fear in individuals. Many false media and false therapies have also been proven to be wrong, and we have all learned about them as a result of mass media coverage. From educating individuals on COVID procedures to social distance, the media plays an important part in the world today. We may also discuss the issues that will arise if the media does not perform its part. An Indonesian investigation discovered that their awareness of preventative measures was limited (Adella Halim et al., 2020). So we may conclude the role of message transmission among individuals. As a result, more emphasis should be placed on educating people rather than focusing on cleaning and maintaining the environment (Rizki et al., 2021).

**Figure 1:**
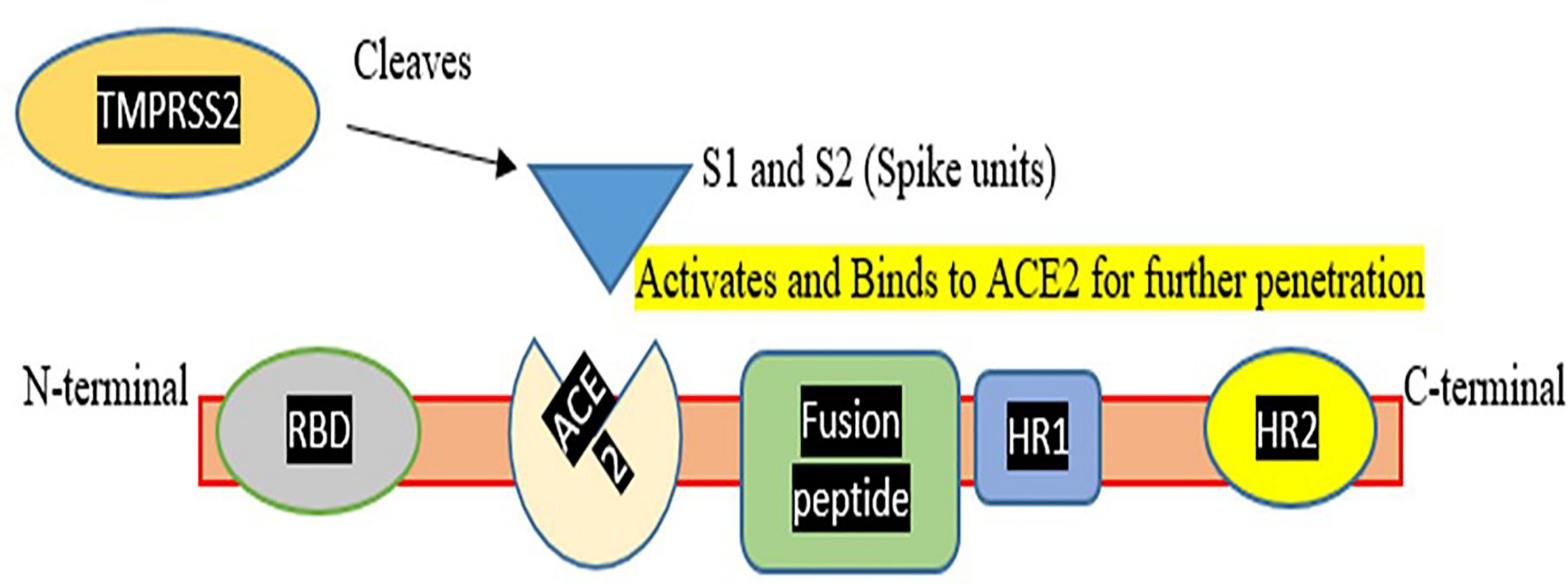
Represents a Corona spike viral fusion protein with N and C terminals, as well as different domains and Spike assembly for hydrolytic cleavage. TMPRSS2, which aids Spike protein activation and penetration, was also demonstrated. This figure demonstrates that how TMPRSS2 cleaves spike protein so that it activates the spike protein for binding to the ACE2 receptor and mediating entry of virus in the host cell.

Various therapies were tested to treat COVID infection which includes: use of probiotics which consists of living microorganisms that benefit human health. Probiotics show a close association with the respiratory system so can be used as a remedy to treat the infection. There are also several pre-existing medicines such as remdesivir, tocilizumab, and other existing and prospective candidates that can be considered for COVID treatment. Several anti-inflammatory like Colchicine and antimalarial drugs such as Hydroxychloroquine and chloroquine (Kumari et al., 2020) were also be tested for the treatment of COVID infection (Asrani et al., 2022). The plant produces secondary metabolites which are generated against viral infection. So considering the fact these metabolites can be targeted using nanoparticles made of carbohydrates and lipids and can be tested for COVID infection (Singh et al., 2021). Therapeutic targets include enzymes involved in viral replication and transcription, helicases and proteases, host cell proteases. Inhibition of host cell endocytosis, anti-sense RNA and ribozyme, neutralizing antibodies, mAbs targeting host cell receptors, or interfering with the S1 RBD domain have been reported (Yong et al., 2019). Focus on the vaccination process is the need of the hour to save lives, as the death rate from COVID-19 is rising every day. To compete with the current situation, more ventilators, oxygen supplies, and ICU beds are needed. However, up to 6.7% of cases might be severe. Patients under the age of one year and those with underlying problems are at a higher risk of developing severe disease. Although several pediatric reports have been published, the epidemiology and clinical patterns of COVID-19, and also treatment options, remain unknown (Tezer and Bedir Demirdağ, 2020). Pregnant women are also COVID vulnerable since there is a decrease in lymphocytes, NKG2A inhibitory receptors, and an increase in ACE2, IL-8, IL-10, and IP-10 during pregnancy, indicating a higher risk of developing COVID-19 (Phoswa and Khaliq, 2020). Various other candidate drugs such as tocilizumab, an immunosuppressive drug, were also tested for COVID-19 use (Asrani and Hassan, 2021; Hariyanto et al., 2021) but did not succeed. A drug named Dexamethasone, a glucocorticoid treatment was also studied, and it was discovered that the death rate was reduced due to its use (Horby et al., 2021). To help and end this pandemic, the production of a safe and efficient vaccine is critical. So, we can either target the Spike protein domain or the RBD domain for vaccine production (refer to **Figure 1**).

The Pfizer-BioNTech COVID-19 mRNA vaccine was the first vaccine approved by the WHO's Strategic Advisory Group of Experts on Immunization (SAGE) as safe and reliable. As we know, healthcare workers are continuously working with COVID patients, so they must be kept in the priority group before vaccinating the population. The COVID-19 vaccine from Pfizer BioNTech has a 95% efficacy against symptomatic SARS-CoV-2 infection. The Janssen Ad26.CoV2.S COVID-19 vaccine was also introduced, which is 85.4% effective against the South African and Brazilian strains of COVID-19. The Oxford/AstraZeneca COVID-19 vaccine (AZD1222) is a non-replicating vector-based vaccine that has been listed for emergency use by the WHO. One version is developed by AstraZeneca-SKBio (Republic of Korea) and the other by the Serum Institute of India (AZD1222). Against symptomatic SARS-CoV-2 infection, this vaccine has a 63.09% efficacy rate. Also in use is the Moderna (mRNA-1273) vaccine. The Moderna vaccine has been shown to protect against COVID-19 with an efficacy of about 92%. The efficacy of the Moderna mRNA vaccine is unaffected by the recent SARS-CoV-2 versions, such as B.1.1.7 and 501Y.V2. Bharat Biotech developed the inactivated BBV152 vaccine with 80% efficacy against COVID-19 (World Health Organization). Sinovac was the first vaccine used in Indonesia against COVID infection (Heriyanto et al., 2021). In Brazil trials, it was identified that Sinovac should be administered in 2 doses and shows the efficacy of 51% against symptomatic SARS-CoV-2 infection and it is 100% effective against severe COVID-19 infection after receiving a 2nd dose (Organization World Health, 2021). Sputnik V has an efficacy rate of 91.6% which has been authorized for use in 70 countries around the world but yet not approved by the U.N. Health (Jones and Roy, 2021). There are also Sinopharm Beijing and Sinopharm Wuhan, both of which have a 79% effectiveness rate against symptomatic COVID strains. The Pfizer vaccine has the greatest efficacy rate of all vaccinations approved by the WHO. The majority of vaccines are based on a buffer dose, which means that two doses must be administered to an individual to develop enough antibodies in the individual's body to protect against COVID infection. Buffer doses play a significant role in COVID vaccinations because they provide a threshold at which enough antibodies are developed to fight COVID infection (Kathy, 2021). To forecast trends in COVID instances, we require an online database to evaluate future predictions or COVID patterns. To find patterns in COVID cases or fatalities with other parameters, the data must be classified into distinct classifiers based on the availability of the data. And, after the parameters are specified, the data in the model must be updated daily (Wang, 2021). This will result in an accurate predictor model. The major goal of the study is to compare the effects of vaccinations on vaccinated and unvaccinated people. This research will conclude on the effectiveness of vaccines and the necessity for a buffer dose to build enough antibodies in a person to protect against COVID infection. By applying numerous machine learning models, we will analyze the accuracy of the model and compute the percentage decrease in COVID cases and deaths as a result of immunizations.

## Materials and Method

### Source of Data

The data used in this work were obtained from the publicly released database ‘Our World in Data’ (Mathieu et al., 2021). The data are publically released and available at www.ourworldindata.org/covid-vaccinations. The data include the number of COVID-19 confirmed cases, number of deaths, and vaccination doses that are given to people in 48 Asian countries from February 24, 2020, to September 26, 2021. However, the database gets updated daily on the website and gives all the information related to the COVID pandemic.

### Data Pre-Processing and Normalization

Data cleaning and pre-processing is a crucial step before starting any analysis as it makes our data reliable for further calculations. Data taken from online databases offer structured data on a day-to-day basis. All variables were converted into monthly data, namely, COVID cases, deaths, and people vaccinated for dosage 1 and dosage 2. The data were normalized to fit it within a range and perform statistical computations using *Standard scaler and min–max scaler*. After data standardization and normalization, logarithmic transformations were also performed so data could become reliable for analysis. Following the data cleaning, statistical calculations were performed using linear, polynomial, OLS regression models and a support vector machine to assess the effect of vaccination over COVID cases and deaths and check the accuracy of the model. All analysis was performed on the Python platform.

### Statistical Analysis

Statistical analysis is essential in verifying assumptions and demonstrating them to create a concrete conclusion about a study. This study focuses on the efficacy of vaccination over COVID cases and COVID deaths. The linear regression analysis will investigate the relevance of vaccinations, followed by polynomial and OLS regression models and SVM models. This will provide information about the effectiveness of being immunized.

### Linear Regression 

In linear regression, two variables are employed: one is the dependent variable (plotted on the y-axis) on which the prediction is based, and the other is an independent variable (plotted on the x-axis) utilized to make the prediction. Variable-based prediction might be univariate (based on one variable) or multivariate (based on several variables) (Moore et al., 2013). A regression line is a straight line that explains how the dependent variable changes with the change in the independent variable. To contrast the model predictions against several sets of field data, we use vaccine dose data to calculate the number of COVID-19 cases and the number of people dying considering the above factors. We fit the model with


(1) Regression line,y=mx + c,

where *c* is an intercept and *m* is the slope and *y* is the dependent variable and *x* is the independent (explanatory) variable ***r^2^*** is the coefficient of determination which is calculated by Karl Pearson’s coefficient (*r*) (Calkins, 2005). This coefficient indicates how many variations are explained by the variable being predicted. The greater the slope, the greater the correlation between the variables, and the greater the ability to explain fluctuations in other variables. Linear regression improves prediction since it focuses on situations with one or more predictor variables (in our study, vaccine data for First Dose and Second Dose) and one outcome variable (Marill, 2004).


(2) Multiple linear regression,y=ax+bz+c,

where *a* and *b* are coefficients of regression, *c* is the intercept while having *x* and *z* as multiple explanatory variables.

The outcome variable, *y*, is a linear function of each predictor variable, *x*, and *z*, forcing the regression model to be a straight line (Marill, 2004). For a good model, the *r-value* should lie in 0.5 to 1.0 so this score gives a good correlation and a good predictor. Regression *analysis* is also used to predict the *p-value* for significance testing. The statistical inference approach is based on a complex network that includes assumptions about how data was gathered and analyzed and how the research results were presented.

### *RMSE* and r^2^-Value

The *RMSE* is the square root mean error. This error value gives an idea about the fitness of the model, i.e., how the values deviate from the true value. *RMSE* is an absolute measure of fit, while *R-squared* is a relative measure of fit. *RMSE* can be interpreted as the standard deviation of the unexplained variance since it is the square root of the variance. It has the advantage of being in the same units as the answer variable. The lower the *RMSE* value the better will be the prediction. If the main goal of the model is prediction, the *RMSE* is the essential criterion for fit because it is a valid standard of how well the model predicts the response.


(3) Mean Square Error=True value−Predicted value

(4) $$Root\;mean\;square\;error={\left(True\;value-Predicted\;value\right)}^{2}$$

We square the error because the estimate can be above or below the true value, resulting in a negative or positive difference. If we didn't square the errors, the sum might fall due to negative differences rather than a strong model fit. Lower values of *RMSE* indicate a better fit.

### Polynomial Regression

For a good predictor model, polynomial regression is the best approach. Some points which do not fit in linear regression fit best in polynomial regression. If the linear regression is underfitting, then we plot polynomial regression to increase complexity in the model by increasing the power of the features and making them new features. All models for degree quadratic, cubic and quartic were constructed using python, and based upon the *RMSE* value, the best was selected. Equation (5) shows a polynomial regression curve of degree 4.


(5) $$\text{Polynomial regression of degree}4,y=x^{4}+\;ax^{3}+\;bx^{2}+\;cx\;+\;d,$$

where, *a*, *b*, and *c* are coefficients of regression and *d* is the intercept. The polynomial curve can be studied for the complexities of COVID cases and deaths based upon vaccination Dosage 1 and Dosage 2.

### OLS Regression Model and P-Value Interpretation

The *p-value *is the “probabilities” of hypotheses. When we perform statistical significance analysis based upon the hypothesis we have designed; if there is a condition in which we have a *p-value* that is very low like 0.0 (although it is not exactly 0), that means that there is a strong correlation between the coefficients and the target (Princeton University). Statistical “significance tests” based on this concept have been a central part of statistical analyses for centuries (Stigler Stephen, 2003). Traditional *p-value* and statistical significance notions have emphasized null hypotheses, treating all other assumptions used to calculate the *p-value* as if they were true. Recognizing that the other assumptions are always suspect, if not outright false, we'll look at the *p-value* in a broader sense as a statistical overview of the compatibility between observed results and what we would expect to see if the entire statistical model (all the assumptions used to compute the *p-value*) were correct. And then, we have an alternate hypothesis in which it is opposite to the null hypothesis. Based on *p-value*, i.e.,

If *p-value* >0.5; so, we accept null hypothesis
If *p-value* <0.5; so we reject null hypothesis

And we can make a concrete statement about our study do have relevance or significance or not. In logical terms, we can say that the *p-value* tests all assumptions related to the model developed, not only focusing on the target hypothesis, i.e., the null hypothesis. OLS regression uses *Student's t distribution* for calculating class intervals from which *p-value* can be interpreted.

To perform the statistical analysis, we can use the OLS regression model, which stands for ordinary least square regression used to compute unknown parameters in the Regression model (linear or polynomial). The method of OLS provides minimum-variance mean-unbiased estimation when the errors have finite variances and are normally distributed. OLS is the maximum likelihood estimator. The (squared) vertical distance from each data point to the line is reduced overall data points by using OLS regression to match a line to bivariate data. The equation:


(6) bOLS=Cov(x, y)/var

describes the slope of this axis *(x)*. As a result, OLS slopes change whether either the way *x* and *y* covary or the variance of the *x*-axis variable changes (Sokal and Rohlf, 2012). This OLS regression calculates a *p-value* which is easy to interpret based on all variables. OLS model was chosen as it gives a reasonable interpretation of models generated and a better way to access the model's relevancy. Using the stats model package, the OLS regression was calculated and the hypothesis generated as stated below:

Null hypothesis = There is no significant effect of people vaccinated over COVID cases and COVID deaths

Alternate hypothesis = There is a significant effect of people vaccinated over COVID cases and COVID deaths.

Using this model, we can be clearer about the significance of vaccinations.

### Support Vector Machine Algorithm

Calculating the accuracy of a model-designed support vector machine (SVM) algorithm is a good measure. It is a supervised learning algorithm that classifies data into 2 classes based upon which training is done and then, using that, future learning classifications are made. These algorithms are more efficient as their performance is high. Using SVM, a hyperplane can be plotted between datasets which are called a decision boundary. Based upon that classifier, classification can be performed. This is an advanced version of the linear and polynomial regression model analyzed above. By using SVM we can make some predictions and these predictions can be compared with the actual values and in the last, the accuracy of the model can also be obtained (Bruno, 2017). SVM regression *analysis* will complete our *analysis* and tell us about the efficacy of the model and decrease the error value of the model by making it more precise.

## Results

### Linear Regression Analysis

**Figure 2:**
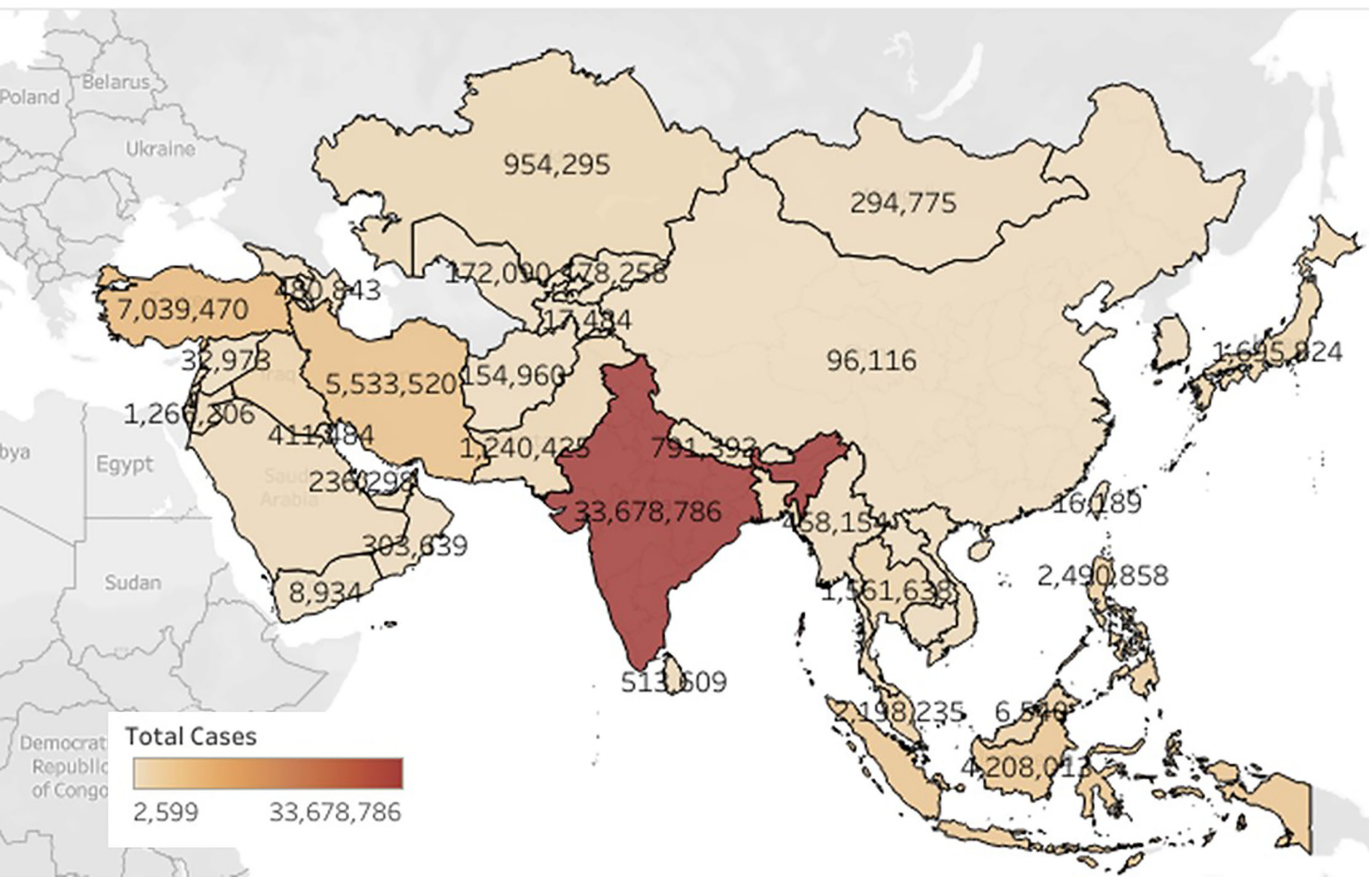
Represents the prevalence of COVID-19 cases in the Asian continent.

The study was done by taking monthly cumulative data of 48 countries from the Asian continent. **Figure 2** is plotted using Tableau (Tableau Software, 2003) software which shows the COVID cases in Asian countries. The color bar designated the incidence of the color scheme based upon the prevalence of COVID cases in a country. The overall COVID cases prediction was performed on the class intervals at 95% confidence and p-value interpreted on for First Dose was 0.0914 [0.087–0.096] (p-value <0.05) and for Second Dose of vaccination was 0.2654 [0.250–0.281] (p-value <0.05). After this, the analysis of the overall death prediction rate on vaccination First Dose was 0.0012 (95% Class interval [0.001–0.001]. Overall death prediction rate on vaccination Second Dose was 0.0034 (95% Class interval [0.003–0.004]. Linear regression analysis revealed the vaccination; these show a positive correlation between variables (vaccination Dose 1 and Dose 2) and target (COVID cases, Deaths). A mathematical model that underpins the approach encapsulates the entire set of assumptions. This model is a statistical representation of data variability, and in theory, it accurately captures all data variability sources. In our model—based upon vaccination doses, i.e., First Dose (partially vaccinated) and Second Dose (fully vaccinated), COVID cases and COVID deaths are calculated and a linear regression analysis was performed. All the models constructed were developed on the python platform. The regression line was plotted for the model designed. Some predictions were performed like if we have 9.5 individuals who were vaccinated for First Dose, 5.5 individuals vaccinated for Second Dose, so we can calculate the decrease in COVID cases and COVID deaths using this linear regression equation and calculating a percentage decrease in the number of cases and deaths upon taking dose 1 and dose 2. For better analysis, the r-value was calculated for each independent variable.

**Figure 3:**
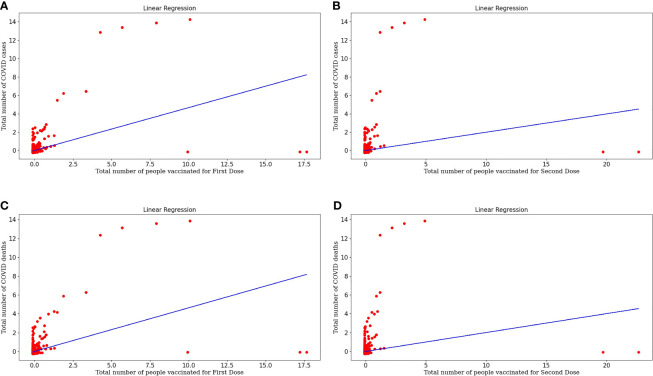
**(A)** Represents linear regression model plotted for Number of COVID-19 cases and Number of people vaccinated for the First Dose. **(B)** Represents linear regression model plotted for Number of COVID-19 cases and Number of people vaccinated for Second Dose. **(C)** Represents linear regression model plotted for Number of COVID-19 deaths and Number of people vaccinated for the First Dose **(D)** Represents linear regression model plotted for Number of COVID-19 deaths and Number of people vaccinated for Second Dose.

**Figure 3A** represents the linear regression model plotted for the number of COVID cases and the number of people vaccinated for the First Dose. This model shows a strong correlation between both the target and variable and the *r-value* is 0.2176. The *RMSE* value is 0.8844 is significantly less, so this is a good predictor. Equation (7) represents the regression line equation for calculating COVID cases based upon the First dose of vaccine. From the regression graph, we can conclude that as the number of vaccination doses increases, there was no effect on COVID cases initially. Still, with time there is a decrease in COVID cases as the graph clearly shows that reaching towards more vaccinated individuals, the cases are decreasing.


(7) $$Number\;of\;COVID\;cases=0.46658304*Number\;of\;people\;vaccinated\;for\;First\;dose+2.46406751e^{-18}$$

**Figure 3B** represents the linear regression model plotted for the number of COVID cases and the number of people vaccinated for the Second Dose. This model shows a good correlation between both the target and variable and the *r-value* is 0.0395 and *RMSE* value is 0.9800, which is less so this model fits well. Equation (8) represents the regression line equation for calculating COVID cases based upon the Second dose of vaccine. From the regression graph, we can conclude that as the number of Second vaccination doses increases, COVID cases decrease. The straight-line plot shows that with an increase in doses, there is a decrease in COVID cases. Also, Dose 2 is more effective in decreasing cases as there are few red spots on the graph, designating more decrement in cases upon taking Dose 2 than Dose 1 of the vaccine because taking the second dose after the first dose generates enough amount of antibodies against COVID infection.


(8) $$Number\;of\;COVID\;cases=0.19890426*Number\;of\;people\;vaccinated\;for\;the\;Second\;dose-1.18585991e^{-17}$$

**Figure 3C** represents the linear regression model plotted for the Number of COVID deaths and the number of people vaccinated for the First Dose. This model shows a good correlation between both the target and variable and the *r-value* is 0.2147 and the *RMSE* value is 0.8861 which is less so this model fits well. Equation (9) represents the regression line equation for calculating COVID deaths based upon the First dose of vaccine. From the regression graph, we can conclude that as the number of vaccination doses increases, there is a decrease in COVID deaths and vaccination plays a critical role in the decrement of COVID cases.


(9) $$Number\;of\;COVID\;deaths=0.46342108*Number\;of\;people\;vaccinated\;for\;First\;dose+1.71500253e^{-17}$$

**Figure 3D** represents the linear regression model plotted for the Number of COVID deaths and the number of people vaccinated for the Second Dose. This model also shows a good correlation between both the target and variable and the *r-value* is 0.0403 and the *RMSE* value is 0.9795 which is less so this is a moderately fitted model. Equation (10) represents the regression line equation for calculating COVID deaths based upon the Second dose of vaccine. From the regression graph, we can conclude that as the number of Dose2 vaccinated individuals increases, there is a huge decrease in deaths. This makes a concluding statement about the effectiveness of vaccinations, especially two doses that act as a buffer against COVID infection.


(10) $$Number\;of\;COVID\;deaths=0.200961*Number\;of\;people\;vaccinated\;for\;Second\;dose+2.97482365e^{-18}$$

### Root Mean Square Error and r-Value Analysis

RMSE is a good measure of how accurately the model predicts the response. RMSE value doesn’t lie in any range but should be less than the sample size. So a lesser RMSE value will be a good fit. **Table 1** shows the RMSE and r-value calculated for each model using a linear regression model.

**Table 1:** Representing *r-value* and *RMSE* error value for all variables used and model designed for a linear regression model.

Model Serial number	Models Designed	*RMSE* value	r^-^value (Variability explained by data)
**1.**	Number of Cases and Dose 1 vaccinated individuals	0.88447	0.2176
**2.**	Number of Cases and Dose 2 vaccinated individuals	0.9800	0.0395
**3.**	Number of Deaths and Dose 1 vaccinated individuals	0.9800	0.2147
**4.**	Number of Deaths and Dose 2 vaccinated individuals	0.9795	0.0403

From the *r-value*, we can say that the data variable explains this percentage of variability and tells us about the relationship between variables.

### Polynomial Regression and P-Value Testing

All models designed above for linear regression have a low mean squared error value. But the linear regression results were not satisfactory to make a concrete decision as some data points are far away from the linear regression line so the polynomial regression was performed for quadratic, cubic, and quartic degrees.

After designing all the degree models for polynomial regression we concluded that the quartic degree polynomial fits better for our model, as it has the lowest *RMSE* value. Also, the resulting polynomial curve can be extrapolated or interpolated to predict death based on the number of people who have been vaccinated. We used this prediction model and generated 70% testing and 30% training data sets using the sklearn package.

**Figure 4:**
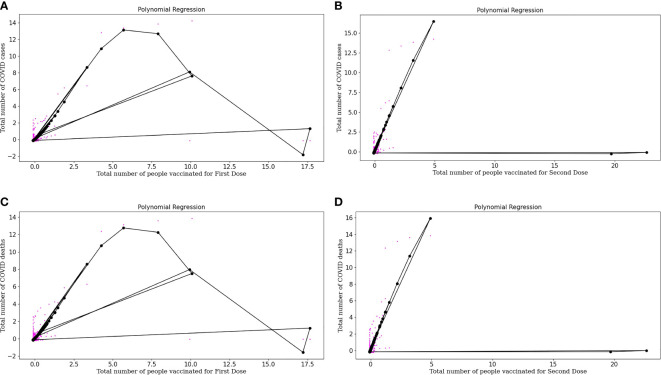
**(A)** Represents Polynomial regression model for quartic degree plotted for People vaccinated for First doses plotted against Number of COVID-19 positive patients. **(B)** Represents Polynomial regression model for quartic degree plotted for People vaccinated for Second doses plotted against Number of COVID-19 positive patients. **(C)** Represents Polynomial regression model for quartic degree plotted for People vaccinated for First doses plotted against Number of COVID-19 deaths. **(D)** Represents Polynomial regression model for quartic degree plotted for People vaccinated for Second Dose (both dose of vaccine) and Number of COVID-19 deaths.

Equation (11) is a polynomial equation that can be used to calculate the number of individuals diagnosed as COVID positive, considering the effect of the first dose of vaccine *(x)*, where 0.04421746 is the intercept and all other multiplications with x are the coefficients of the polynomial equation. Also, **Figure 4A** represents the polynomial regression model for quartic degree plotted for people vaccinated for First Dose plotted against the number of COVID positive patients. From the above polynomial regression curve, we can identify a pattern about vaccinations, i.e., initially, we can see more data sets, i.e., more COVID cases and there were no vaccinations available. But with the time the vaccinations started, and then there was a decrease in cases with an increase in vaccinations as with time we can see a decrease in color spots in the regression curve plotted.


(11) $$Number\;of\;COVID\;positive\;patients=1.55622435*x^{4}+0.63234385*x^{3}-0.11061507*x^{2}+0.00396629*x+0.04421746$$

Equation (12) is a polynomial equation that can be used to calculate the number of individuals diagnosed as COVID positive, considering the effect of the second dose of vaccine *(x)*, where 0.16024788 is the intercept and all other multiplications with *x* are the coefficients of the polynomial equation. **Figure 4B** represents the polynomial regression model for quartic degree plotted for people vaccinated for the Second dose of vaccine plotted against the number of COVID positive patients. Polynomial regression curves are more reliable than linear regression curves as they consider the complexities of variables. From the regression, curve plotted we can conclude that with an increase in Dose 2, there is a sharp decrease in COVID cases which tells about the effectiveness of the buffer dose after the first dose.


(12) $$Number\;of\;COVID\;positive\;patients\;upon\;getting\;both\;vaccine\;doses=3.60870165e^{+00}*x^{4}+7.78207554e^{-02}*x^{3}-3.20635017e^{-02}*x^{2}+9.52339532e^{-04}*x+0.16024788$$

Equation (13) is a polynomial equation that can be used to calculate the number of individuals who died considering the effect of the first dose of vaccine.


(13) $$Number\;of\;people\;died\;due\;to\;COVID\;upon\;taking\;Dose1=1.80295049*x^{4}+0.51069827*x^{3}-0.09657263*x^{2}+0.00351539*x+0.06844712$$

where *x* is the number of vaccinated people partially with a complex of power 4 and 0.06844712 is the intercept and all other multiplications with *x* are the coefficients of the polynomial equation. **Figure 4C** represents the Polynomial regression model for quartic degree plotted for people vaccinated for First Dose plotted against the number of COVID deaths. Polynomial regression curve plotted for COVID deaths and vaccination doses, we can conclude that with an increase in Dose 1, COVID deaths decrease.


(14) $$Number\;of\;people\;died\;due\;to\;COVID\;upon\;taking\;Dose2=3.76222600e^{+00}*x^{4}-8.88558776e^{-03}*x^{3}-2.48292596e^{-02}*x^{2}+7.89026296e^{-04}*x+0.17096195$$

Equation (14) is a polynomial equation that can calculate the number of deaths considering the effect of dose 2, where *x* is the number of people who are entirely vaccinated with a complex of power 4 and 0.17096195 is the intercept and all other multiplications with *x* are the coefficients of a polynomial equation. **Figure 4D** represents the polynomial regression model for quartic degree plotted for people vaccinated for Second Dose (both doses of vaccine) and the number of COVID deaths. From the regression curve plotted, we can conclude that with an increase in Dose 2, there is a sharp decrease in COVID deaths which tells us about the effectiveness of buffer dose.

After fitting the curve for degree 4 the prediction was made based upon the number of people vaccinated. Based upon the above problem and the prediction model developed, we predicted the number of people who are diagnosed COVID-19 positive and also who died based upon the vaccination doses received. **Table 2** represents the *RMSE* and r-values for the models designed regarding COVID cases and COVID deaths for the First Dose and Second Dose vaccinated individuals. If 9.5 people are vaccinated for First Dose, then the number of people who die due to COVID will be = 4.4020 number of people and when some people received their Second Dose, the number of people who died is reduced to 1.1058.

**Table 2:** Representing *r-value* and RMSE error value for all variables used in the polynomial regression model.

Model Serial number	Model Designed	Degree of polynomial Fitted best	*RMSE* value	r-value
**1.**	Number of Cases and Dose 1 vaccinated individuals	Degree 4	0.4678	0.7811
**2.**	Number of Cases and Dose 2 vaccinated individuals	Degree 4	0.5270	0.7221
**3.**	Number of COVID deaths and Dose 1 vaccinated individuals	Degree 4	0.4884	0.7614
**4.**	Number of COVID deaths and Dose 2 vaccinated individuals	Degree 4	0.5379	0.7106

Upon estimating this we also predicted the number of cases, i.e., they are decreasing upon vaccination. So, an *analysis* was made on the prediction of COVID cases. We found that if 9.5 people are vaccinated for First Dose, then the upcoming cases were 4.43 and if they are vaccinated for Second Dose, these cases will reduce to 1.093. So this concludes that upon vaccination rate of COVID-19 infection will also reduce to 75.319% and the COVID death rate will also reduce to 74.89%.

As a result of this estimate, we can conclude that vaccination is necessary to reduce the death risk and the infection rate, so more vaccination centers should be established for people to be vaccinated. From the above analysis, we can make a concrete statement about the necessity of vaccination. After this, we can perform *OLS regression* for better understanding and hypothesis testing. Polynomial regression model revealed about the COVID cases which reduced up to 74.89% upon vaccination and death rate which reduced to 75.31% after receiving the Second dose of vaccination which is a great decrease in death number which makes a concrete statement that being vaccinated decreases the risk of death due to COVID. The null hypothesis was rejected after analyzing the *p-value* using the OLS regression algorithm as the *p-value* (0.05) >0.00. We can say there is a significant effect of vaccination over COVID cases and death numbers. Using *Student's t distribution* test, the *p-value* was calculated. This tells us about the behavior of the sample mean on the number of observations. Using the OLS regression model using a 95% confidence interval, the class interval was checked. The coefficients lie between these confidence intervals, so we can say that the *p-value* is either 0.05 or 0 so the OLS regression model *p-value* was calculated. It is 0.00, which is less than 0.05, so the null hypothesis is rejected. We can say there is a significant effect of people vaccinated over non-vaccinated for COVID death and cases. This whole study shows that after being vaccinated for both, the Doses of vaccine, the death rate, and COVID cases will decrease, making a significant difference. The accuracy of the models used may be measured by overlaying the predicted and actual datasets, and for this, a more advanced regression approach, such as Support Vector Regression, will be used for further analysis.

### Support Vector Regression Analysis

Using SVM, the accuracy of the model was tested and it showed 61.45 and 67.96% accuracy rates for COVID cases and COVID deaths over Dosage 1 and Dosage 2 vaccinated individuals, respectively, which is a good performance score for the model designed. **Table 3** represents the *RMSE* error value and the *r-value* calculated for the model.

**Table 3:** Representing *r-value* and *RMSE* error value for all variables used in the support vector regression model.

Model Serial number	Model Designed	*RMSE* value	*r-value*
**1.**	Number of Cases and Dose 1 vaccinated individuals	0.4051	0.4537
**2.**	Number of Cases and Dose 2 vaccinated individuals	0.4746	0.2496
**3.**	Number of COVID deaths and Dose 1 vaccinated individuals	0.4772	0.4743
**4.**	Number of COVID deaths and Dose 2 vaccinated individuals	0.5402	0.2810

The SVM model is best in all the models discussed above, and the root means the squared error is also reduced. This makes our model more reliable. To verify the performance of the model created using SVM accuracy rate calculation is the most crucial step. The accuracy rate of the model was calculated for 30% testing datasets and 70% training datasets and it was found that the model generated using SVM for Prediction of COVID cases and deaths corresponding to Dosage 1 vaccinated population was 61.45% and for Prediction of COVID cases and deaths corresponding to Dosage 2 vaccinated population was 67.96%. This accuracy rate is excellent and we can conclude that our Support Vector Machine model shows good performance and accurate results.

**Figure 5:**
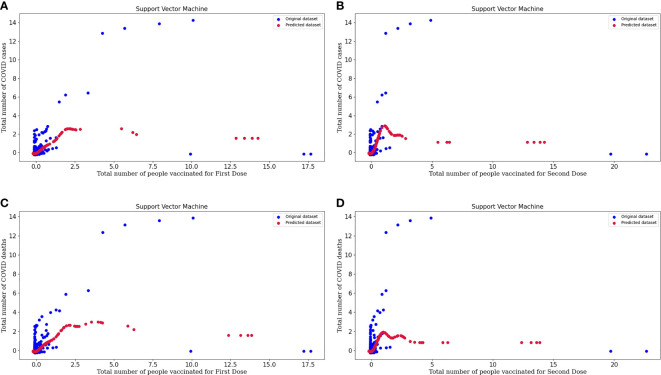
**(A)** Represents the Support vector machine model for People vaccinated for the First Dose and the Number of COVID-19 cases. **(B)** Represents the Support vector machine model for People vaccinated for the Second Dose and the Number of COVID-19 cases. **(C)** Represents the Support vector machine model for People vaccinated for the First Dose and the Number of COVID-19 deaths. **(D)** Represents the Support vector machine model for People vaccinated for the Second Dose and the Number of COVID-19 deaths.

**Figure 5A** represents the support vector machine model for people vaccinated for the First Dose and the number of COVID cases. In the above graph, we can study the original and predicted dataset pattern by considering the spots on the plot.

**Figure 5B** represents the support vector machine model for people vaccinated for the Second Dose and the number of COVID cases. From the plot, we can conclude about the model's efficacy, and the predicted dataset is following the slightly same pattern as the points of the original dataset. So, this tells us about the excellent performance of the model.

**Figure 5C** represents the support vector machine model for people vaccinated for the First Dose and the number of COVID deaths.

**Figure 5D** represents the support vector machine model for people vaccinated for the Second Dose and the number of COVID deaths. In these plots, original datasets and predicted datasets are plotted in blue and crimson colors, respectively. As the performance of the model is high, our predictor model is good and there is an effect of vaccinations over the decrease in the rate of COVID cases and deaths. By plotting to scatter plots for support vector regression, we can conclude that our predicted datasets and original datasets are overlapping. Hence, we can say that our predicted dataset is good. SVM regression is more accurate and also decreases the error value. Support vector regression makes the model more reliable and stable.

### Performance of All the Models Analyzed

We have used Linear Regression, Polynomial Regression, and Support Vector Machine algorithms to analyze the COVID data and make concluding remarks. Initially, in linear regression results, we observed that some data points are far away from the regression line. However, when we drew a polynomial curve, the findings revealed that all data points were considered, resulting in a more impactful analysis. **Table 4** below represents the *RMSE* value and *r-value* calculated for each model using different machine learning algorithms. After analyzing all models designed using different machine learning algorithms, we identified that SVM is the best of all and it gives more accurate results. The *RMSE* error value is minimum and hence, the performance of the model is high.

**Table 4:** Represents the comparison of *r-value* and *RMSE* error value for all models analyzed using a different machine learning algorithm.

Models	Linear Regression		Polynomial Regression		Support Vector Machine	
	*RMSE* value	*r-value*	*RMSE* value	*r-value*	*RMSE* value	*r-value*
Number of Cases and Dose 1 vaccinated individuals	0.88447	0.2176	0.4678	0.7811	0.4051	0.4537
Number of Cases and Dose 2 vaccinated individuals	0.9800	0.0395	0.5270	0.7221	0.4746	0.2496
Number of deaths and Dose 1 vaccinated individuals	0.9800	0.2147	0.4884	0.7614	0.4772	0.4743
Number of deaths and Dose 2 vaccinated individuals	0.9795	0.0403	0.5379	0.7106	0.5402	0.2810

## Discussion

People will benefit from such a model by lowering their risk of COVID-19 infection, which reduces to 75.31 and a 74.89% decrement in the death rate on getting fully vaccinated. We may argue that prediction is constrained since we used data from an online database that is not unique to a patient but is a summary of everyday COVID events. We might have stronger predictions if we had person-level data outside the scope of the work we discussed above. Various cells in the data are null, or data might not be available for that day, making interpretation difficult. As the pandemic progresses, this model will require an update, recalibration, and redesigning. Our study isn't meant to evaluate the genuine issue of COVID cases and vaccination, which would necessitate a community-based approach to healthcare delivery research, which is not possible due to data limitations. As we all know, certain vaccinations do not require a buffer dose of vaccination while others do. To ensure data reliability, only Asian countries were included in the analysis, because the majority of Asian countries employ buffer dose vaccines. Other factors such as lockdowns, social distancing, and improved healthcare facilities might also significantly affect the death rate. Still, these factors have not been included in the present study.

## Conclusion

A predictor model is being developed that calculates the number of people getting infected for COVID-19 upon taking vaccinations and who died due to COVID-19 based upon the vaccination doses. This estimate is based on people who received their first or both doses of vaccine and can conclude the need for vaccination. The vaccination process has started is going on in the world currently. We can consider two types of population: one that has encountered COVID-19 and the other that is not COVID positive. There are numerous challenges with getting vaccinated nowadays. Therefore we may divide our population into three groups: those who have got First Dose, those who delay taking Second Dose, and those who have already got the Second Dose. The immunization panel stated that a COVID patient develops antibodies naturally over 100–180 days. They are immune and should be vaccinated after 6 months of recovery. The population should be vaccinated since the body will generate an immune response after the first dosage, increasing immunogenicity and vaccination efficacy. We may also claim that Second Dose is required since it will produce a sufficient level of antibody response in our bodies, allowing patients to fight infection more effectively than non-vaccinated people. Nowadays, the second buffer dose duration time is increased by the WHO and also by the institutes which are developing a vaccine as if the second dose is taken after an interval of 6 to 8 weeks, then it is more efficient. Now it has been approved according to the United Kingdom COVID-19 working group. The dosing interval can be increased to 12–16 weeks. Also, a study published in an international medical journal reported that we should increase the dosing interval of the vaccine. This interval will increase the efficacy of the vaccine from 55.1 to 81.3%, which is quite higher in building immune response (Calisher et al., 2020). It was also reported that receiving the first dose of vaccine raises immunity by 64% while receiving the second dosage raises immunity by 94% (Centre of Disease Control and Prevention). Vaccination is critical, especially both dosages, to build enough immunological response in a person's body to render them immunity against COVID-19 infection. People will benefit from such a model by lowering their risk of COVID-19 infection, which reduces to 75.31% on getting fully vaccinated and the death rate. We have demonstrated a 75.31% decrease in COVID cases and a 74.89% decrement in the death rate upon getting fully vaccinated. After analyzing various machine learning algorithms in the above study, we identified that the high SVM model performance predicts more accurate results. As a result, we can make a clearer statement regarding the necessity for vaccines, and we can recommend that the number of vaccine doses and vaccination facilities must be increased. We must overcome the inevitability of a shortage of funding, vaccine output, and inequity in health care. Vaccination is critical, especially both dosages, to build immunity against COVID-19 infection and lower the risk of death.

## Data Availability Statement

Data used for this article are available at: www.ourworldindata.org/covid-vaccination.

## Author Contributions

VR, IKS, and AS conceived and designed research. VR, MB, T, PS, RA, MFA, AH, MIH, IKS, and AS collected and analyzed the data. VR, MB, IKS, MFA, PS, RA, AH, AS, and MIH, wrote the manuscript. All authors contributed to the article and approved the submitted version.

## Conflict of Interest

The authors declare that the research was conducted in the absence of any commercial or financial relationships that could be construed as a potential conflict of interest.

## Publisher’s Note

All claims expressed in this article are solely those of the authors and do not necessarily represent those of their affiliated organizations, or those of the publisher, the editors and the reviewers. Any product that may be evaluated in this article, or claim that may be made by its manufacturer, is not guaranteed or endorsed by the publisher.
